# Preclinical *in vitro* screening of newly synthesised amidino substituted benzimidazoles and benzothiazoles

**DOI:** 10.1080/14756366.2020.1850711

**Published:** 2021-01-06

**Authors:** Livio Racané, Maja Cindrić, Ivo Zlatar, Tatjana Kezele, Astrid Milić, Karmen Brajša, Marijana Hranjec

**Affiliations:** a Department of Applied Chemistry, Faculty of Textile Technology, University of Zagreb, Zagreb, Croatia; b Department of Organic Chemistry, Faculty of Chemical Engineering and Technology, University of Zagreb, Zagreb, Croatia; c Pharmacology in vitro, Fidelta Ltd, Zagreb, Croatia; d DMPK, Fidelta Ltd, Zagreb, Croatia

**Keywords:** Amidines, benzimidazoles, benzotiazoles, 2D and 3D in vitro cytotoxicity assay, apoptotic activity, ADME

## Abstract

Newly synthesised benzimidazole/benzotiazole derivatives bearing amidino, namely 3,4,5,6-tetrahydropyrimidin-1-ium chloride, substituents have been evaluated for their potential antitumor activity *in vitro*. Compounds and standard drugs (doxorubicin, staurosporine and vandetanib) were tested on three human lung cancer cell lines A549, HCC827 and NCI-H358. We tested compounds in MTS citotoxicity assay and in BrdU proliferative assay performed on 2 D and 3 D assay format. Because benzmidazole scaffold is similar to natural purines, we tested the most active compounds for ability to induce cell apoptosis of A549 by binding to DNA in comparison with doxorubicin and saturosporine. Additionally, the ADME properties of the most active benzothiazole/benzimidazole and non-active compounds were determined to see if the different ADME properties are the cause of different activity in 2 D and 3 D assays, as well as to see if the tested active compounds have drug like properties and potency for further profilation. ADME characterisation included solubility, lipophilicity, permeability, metabolic stability and binding to plasma proteins. In general, the benzothiazole derivatives were more active in comparison to their benzimidazole analogues. The exception was 2-phenyl substituted benzimidazole **6a** being active with very pronounced activity especially towards HCC827 cells. All active compounds have similar mode of action on A549 cell line as standard compound doxorubicin, which binds to nucleic acids with the DNA double helix. Tested active benzothiazole compounds were characterised by moderate to good solubility, good metabolic stability, low permeability and high binding to plasma proteins. One tested active benzimidazole derivative showed ADME properties, but lower lipophilicity resulted in low PPB and higher metabolic instability. In addition, no significant difference was observed in ADME profile between active and non-active compounds.

## Graphical Abstract

**Figure:**
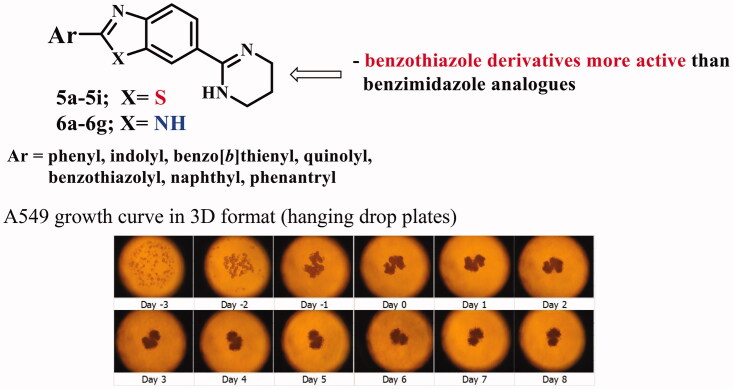

## 1. Introduction

Lung cancer is the second most common cancer (about 14%) of new diagnosted cancers^1^. Although new drugs and approaches to treating lung cancer have been discovered in the last five years, chemotherapeutics are still the first line of therapy. Despite good patient response, there is still a medical need for new chemotherapeutics, primarily due to cancer drug resistance and toxicity, which are the main therapy limitations of the efficacy and clinical outcomes.

Taking into account the great biological importance of benzimidazole and benzothiazole derived natural, semisynthetic or synthetic derivatives as well as their versatile pharmacological features, these nitrogen scaffolds become unavoidable structural motifs in the rational design of novel drugs^2–6^. Nowadays there is still an increasing interest in medicinal and pharmaceutical chemistry for incorporation of benzimidazole/benzothiazole highly-privileged building substructures in order to developed novel heterocycles with possible pharmacological, chemical or industrial applications^7^^,^^8^. Suchlike derivatives display a broad spectrum of different biological features such as anticancer, antiviral, antioxidant, antibacterial, antifungal, antihistaminic, anti-inflammatory, *etc*^9–11^. Furthermore, the structural similarity of benzimidazole scaffold with naturally occurring purines is of great importance for studying the role of prepared derivatives in the function of many biologically important molecules like DNA, RNA or different proteins in living organisms^12^^,^^13^.

Additionally, the literature review revealed that amidines are structural parts of numerous biologically active compounds like many important medical and biochemical agents^14^. Recently, we have published several papers regarding the amidino substituted benzimidazole/benzotiazole derivatives with amidine group as positively charged substituent, placed at the end of the heteroaromatic substructures^15^. We have proven that within designing suchlike derivatives, the biological activity could be significantly improved while many of derivatives showed interaction with an electronegatively charged biological molecule such as DNA. The synthesised derivatives displayed antiproliferative, antibacterial, antifungal and antioxidative activity with several active compounds which were chosen as lead compounds for further optimisation and modification to get more active, selective and less cyctotoxic derivatives with improved physico-chemical properties^16^. Recently, we have published several papers describing the antiproliferative activity of various benzothiazole and benzimidazole derivatives substituted with either carboxamido, amino, halogeno, cyano, amidino, amino or nitro groups placed at different positions on the mentioned scaffold^17–20^. The most significant biological importance was observed with amidino substituted benzazoles bearing different types of amidine substituents suchlike unsubstituted, isopropyl, morpholinyl or imidazolinyl. Obtained results revealed that among all synthesised benzazole derivatives, cyclic amidino substituent, namely 2-imidazolinyl group showed the most significant influence and the enhancement of the antiproliferative activity *in vitro* with with IC_50_ values in submicromolar range of concentrations^14^^,^^21^. Very recently, we present the design and synthesis of amidino substituted 2-phenylbenzothiazole and benzimidazole derivatives with the variable number of hydroxy and methoxy groups attached to the phenyl ring and explore their antiproliferative and antioxidative activity *in vitro*^21^. Particularly, we were interested in synthesis and antiproliferative activity screening of cationic diamidino-substituted derivatives of phenylbenzothiazolyl and dibenzothiazolyl furans and thiophenes^15^, bisbenzothiazolyl-pyridines and pyrazine^22^, phenylene-bisbenzothiazoles^23^, amidino substituted 2-arylbenzothiazole hydrochloride^24^ and mesylate^25^.

Within this work, as a continuation of our previous scientific work, herein we present the synthesis and preclinical screening *in vitro* on human lung cancer cells of novel amidino substituted benzimidazole/benzothiazole derivatives substituted with different aryl moieties in position 2 of benzazole scaffold.

As we have describe previously^26^, two-dimensional (2 D) cell cultures are not able to imitate complex tumour structure as three-dimensional (3 D) cell cultures. Also, 3 D techniques have already great impact in screening of active new chemical entities (NCE) with potential antitumor activity. Among various 3 D methods we developed screening on 3 D spheroids because is more likely to tumour growth and physiology. Cytotoxicity and proliferation assays reads out give as two distinct characteristics of cells^27^, therefore we tested compounds in MTS cytotoxicity assay and in BrdU proliferative assay performed on 2 D and 3 D assay format. For 2 D cell assay format, we used a classic two-dimensional *in vitro* assay^28^ and as 3 D assay we used a hanging drop proliferation cell assay, previosuly described^29^.

Mechanism of action of most active compounds in antiproliferative assay was tested measuring apoptosis (anexin V staining) by FACS analysis in comparison with doxorubicin and staurosporine.

ADME properties are dependent of structural characteristics of newly synthesised molecules. Different modifications are undertaken and tested during early drug discovery in order to achieve better and improved drug-like properties^30^. ADME characterisation represents an important step in the drug discovery process and it includes several *in vitro* assays covering physicochemical and biochemical properties such as solubility, lipophilicity, permeability, metabolic stability and binding to plasma proteins^31^. Here, we have evaluated major ADME properties to see if compound’s activity, obtained in 3 D cell cultures, is the consequence of different physicochemical and biochemical properties in comparison with non-active compounds, as well as to see if compounds have drug-like properties and potential for further profiling in *in vivo* models.

**Scheme 1.**
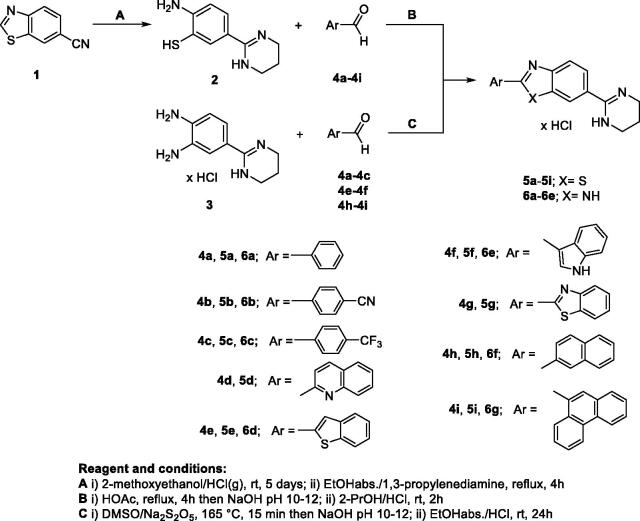
Synthesis of amidino-substituted benzazoles.

Compounds selected for ADME characterisation (Table 3) included five active derivatives from benzothiazole series **5c–5e, 5 g, 5 h** (Scheme 1) and one active benzimidazole **6a** (Scheme 1), respectively. To test pannel, we also added 7 non-active benzimidazole/benzothiazole derivatives.

**Table 3.** Summary of ADME properties of active compounds.

	5c	5d	5e	5g	5h	6a
Kinetic solubility range afer 2 h (µM)	>100	30–100	30–100	30–100	10–30	>100
Chrom logD	3.31	2.87	3.19	2.71	3.31	0.92
Microsomes (1 µM) Predicted in vivo hep CL (%LBF)						
Mouse	<30	<30	63	<30	<30	41
Human	<30	<30	<30	<30	<30	56
PPB % bound (recovery)						
Mouse	97.5 (86)	96.0 (82)	98.7 (82)	96.0 (85)	97.3 (85)	62.9 (79)
Human	97.0 (89)	94.7 (76)	97.9 (79)	95.1 (94)	97.6 (80)	59.7 (75)
Plasma stability (%remaining at 4 h)						
Mouse	94	85	77	81	85	79
Human	83	81	82	86	74	73
MDCKII-MDR1						
Papp(A2B),	<0.1 – >6.6	<0.1 – >6.5	<0.1 – >2.0	<0.1 – >3.9	<0.1 – >0.7	0.4 – >0.7
Papp(B2A),	21.5 – >5.8	15.8 – >5.6	7.6 – >1.8	16.5 – >5.7	7.7 – >1.8	1.4 – >0.9
Efflux ratio (w/o-> w/ P-gp inhibitor)	>200 – >0.9	>158 – >0.9	>78 – >1.0	>17 – >2.0	>77 – >3.5	3.7 – >1.5

## 2. Experimental part

### 2.1. Chemistry

Melting points were determined by means of Original Kofler Mikroheitztisch apparatus (Reichert, Wien). The ^1^H NMR and the ^13 ^C NMR spectra were recorded with the Bruker Avance DPX-300 or Bruker AV-600 using TMS as internal standard. Chemical shifts are reported in parts per million (ppm) relative to TMS. Elemental analyses for carbon, hydrogen and nitrogen were performed on Perkin-Elmer 2400 elemental analyser. Analyses are indicated as symbols of elements, analytical results obtained are within 0.4% of the theoretical value. All compounds were routinely checked by TLC using Merck silica gel 60 F-254 glass plates.

Synthesis of 6-cyanobenzothiazole (**1**) was carried out according to the literature^32^. Synthesis of 2–(3,4-diaminophenyl)-3,4,5,6-tetrahydropyrimidin-1-ium chloride (**3**) was carried out according to the literature^14^.

#### 2.1.1. Synthesis of 2-amino-5–(3,4,5,6-tetrahydropyrimidin-1-ium-2-yl)benzenethiolate 2

A suspension of 6-cyanobenzothiazole **1** (4.0 g, 25 mmol) in 50 ml of dry 2-methoxyethanol was cooled to 5 °C and saturated with dry gaseous HCl. The flask was stoppered and stirred at room temperature for 4 days. Excess of HCl was removed from the suspension with a stream of nitrogen and the reaction mixture was poured into diethyl-ether. The resulting solid was filtered off, washed with diethyl-ether and dried under reduced pressure over KOH. The intermediate imidoyl-ether dihydrochloride was suspended in 100 ml of abs ethanol and 1,3-propylenediamine (10.5 ml, 125 mmol) added under nitrogen atmosphere.

The reaction mixture was refluxed for 4 h, cooled under nitrogen to 5 °C, and the obtained solid was filtered off and washed with dry ether. The solid mixture was suspended and heated to boiling in 70 ml of deoxygenated water, cooled under nitrogen at 5 °C for 2 h, and the resulting precipitate was filtered off and dried under reduced pressure over KOH. Yield of pure compound **2** as pale yellow solid was 4.26 g (83.2%) mp = 284–288 °C. ^1^H NMR (300 MHz, TFA-*d*_1_) *δ* 8.19 (bs, 2H), 7.97 (d, *J* = 1.8 Hz, 1H), 7.76 (d, *J* = 8.4 Hz, 1H), 7.70 (dd, *J* = 2.0 Hz, *J* = 8.4 Hz, 1H), 3.69 (t, *J* = 5.6 Hz, 4H), 2.19 (m, 2H); ^13 ^C NMR (75 MHz, HOAc-*d*_4_) *δ* 159.1, 152.6, 133.1, 127.7, 116.3, 114.3, 112.1, 39.1, 18.2; Analysis calcd for C_10_H_13_N_3_S: C, 57.94; H, 6.32; N, 20.27% Found: C, 57.72; H, 6.54; N, 20.31%.

#### 2.1.2. General method for preparation of compounds 5a–5i

To a stirred solution of 2-amino-5–(3,4,5,6-tetrahydropyrimidin-1-ium-2-yl)benzenethiolate **2** (0.104 g, 0.5 mmol) in glacial acetic acid (5 ml), a corresponding carbaldehyde **4a**–**4i** (0.5 mmol) was added under nitrogen atmosphere and heated to reflux for 4 h. The reaction mixture was poured onto ice and made alkaline with 20% NaOH. The resulting free base was filtered off, washed with water, and dried. The free base was suspended in 2-propanole and concd HCl (84 µl, 1.0 mmol) was added. The reaction mixture was stirred at room temperature for 1–2 h and cooled in refrigerator overnight. The resulting precipitate was filtered off, washed with diethyl-ether, and dried at 75 °C.

##### 2.1.2.1. 2-Phenyl-6–(3,4,5,6-tetrahydropyrimidin-1-ium-2-yl)benzothiazole chloride 5a

Compound **5a** was prepared from benzaldehyde **4a** (0.053 g, 0.5 mmol). The resulting free base was converted into salt as described below to obtained 0.064 g (38.8%) of white powder; m.p. = 272–275 °C; ^1^H NMR (300 MHz, DMSO-*d*_6_) *δ* 10.23 (s, 2H), 8.63 (d, *J* = 1.6 Hz, 1H), 8.28 (d, *J* = 8.6 Hz, 1H), 8.20 − 8.13 (m, 2H), 7.88 (dd, *J* = 1.8 Hz, *J* = 8.6, Hz, 1H), 7.68 − 7.58 (m, 3H), 3.54 (m, 4H), 2.01 (m, 2H); ^13 ^C NMR (151 MHz, DMSO-d_6_) *δ* 171.2, 158.8, 156.1, 134.6, 132.3, 132.2, 129.5 (2 C), 127.5 (2 C), 126.0, 125.3, 123.0, 122.8, 38.8 (2 C), 17.7; Analysis calcd for C_17_H_16_ClN_3_S: C, 61.90; H, 4.89; N, 12.74%; Found: C, 61.73; H, 5.11; N, 12.90%.

##### 2.1.2.2. 2–(4-Cyanophenyl)-6–(3,4,5,6-tetrahydropyrimidin-1-ium-2-yl)benzothiazole chloride 5 b

Compound **5 b** was prepared from 4-cyanobenzaldehyde **4 b** (0.067 g, 0.5 mmol). The resulting free base was converted into salt as described below to obtained 0.101 g (57.1%) of coloreless powder; m.p. >300 °C; ^1^H NMR (300 MHz, DMSO-*d*_6_) *δ* 10.27 (s, 2H), 8.69 (d, *J* = 1.6 Hz, 1H), 8.36–8.33 (m, 3H), 8.09 (d, *J* = 8.5 Hz, 2H), 7.92 (dd, *J* = 1.8 Hz, *J* = 8.6 Hz, 1H), 3.53 (m, 4H), 2.02 (m, 2H); ^13 ^C NMR (75 MHz, DMSO-d_6_) *δ* 169.2, 158.7, 155.8, 136.0, 135.0, 133.4 (2 C), 128.2 (2 C), 126.3, 125.9, 123.5, 123.2, 118.2, 114.0, 38.8 (2 C), 17.7; Analysis calcd for C_18_H_15_ClN_4_S: C, 60.92; H, 4.26; N, 15.79%; Found: C, 60.81; H, 4.23; N, 15.88%.

##### 2.1.2.3. 2–(4-Trifluoromethylphenyl)-6–(3,4,5,6-tetrahydropyrimidin-1-ium-2-yl)benzothiazole chloride 5c

Compound **5c** was prepared from 4-trifluoromethylbenzaldehyde **4c** (0.087 g, 0.5 mmol). The resulting free base was converted into salt as described below to obtained 0.107 g (53.8%) of white powder; m.p. = 279–283 °C; ^1^H NMR (300 MHz, DMSO-*d*_6_) *δ* 10.26 (s, 2H), 8.69 (d, *J* = 1.6 Hz, 1H), 8.38 (d, *J* = 8.1 Hz, 2H), 8.34 (d, *J* = 8.6 Hz, 1H), 7.99 (d, *J* = 8.3 Hz, 2H), 7.92 (dd, *J* = 1.9 Hz, *J* = 8.6 Hz, 1H), 3.54 (t, *J* = 5.6 Hz, 4H), 2.01 (m, 2H); ^13 ^C NMR (75 MHz, DMSO-d_6_) *δ* 169.4, 158.7, 155.9, 135.8, 134.9, 131.5, 128.4 (2 C), 126.4 (2 C), 126.3, 125.8, 123.8, 123.4, 123.1, 38.4 (2 C), 17.7; Analysis calcd for C_18_H_15_ClF_3_N_3_S: C, 54.34; H, 3.80; N, 10.56%; Found: C, 54.22; H, 3.85; N, 10.66%.

##### 2.1.2.4. 2-(Quinoline-2-yl)-6–(3,4,5,6-tetrahydropyrimidin-1-ium-2-yl)benzothiazole chloride 5d

Compound **5d** was prepared from quinoline-2-carbaldehyde **4d** (0.079 g, 0.5 mmol). The resulting free base was converted into salt as described below to obtained 0.121 g (63.7%) of coloreless powder; m.p. >300 °C; ^1^H NMR (300 MHz, DMSO-*d*_6_) *δ* 10.22 (s, 2H), 8.69–8.66 (m, 2H), 8.50 (d, *J* = 8.5 Hz, 1H), 8.38 (d, *J* = 8.6 Hz, 1H), 8.19–8.13 (m, 2H), 7.96 − 7.88 (m, 2H), 7.76 (m, 1H), 3.54 (t, *J* = 4.8 Hz, 4H), 2.02 (m, 2H); ^13 ^C NMR (75 MHz, DMSO-d_6_) *δ* 172.8, 158.8, 158.7, 156.3, 149.9, 147.1, 138.2, 135.7, 131.0, 128.9, 128.3, 126.0, 123.7, 123.1, 118.0, 38.9 (2 C), 17.7; Analysis calcd for C_20_H_17_ClN_4_S: C, 63.07; H, 4.50; N, 14.71%; Found: C, 62.99; H, 4.51; N, 14.78%.

##### 2.1.2.5. 2-(Benzo[b]thiophen-2-yl)-6–(3,4,5,6-tetrahydropyrimidin-1-ium-2-yl)benzothiazole chloride 5e

Compound **5e** was prepared from benzo[*b*]thiophene-2-carbaldehyde **4e** (0.081 g, 0.5 mmol). The resulting free base was converted into salt as described below to obtained 0.120 g (62.2%) of white powder; m.p. >300 °C; ^1^H NMR (300 MHz, DMSO-*d*_6_) *δ* 10.23 (s, 2H), 8.62 (d, *J* = 1.6 Hz, 1H), 8.40 (s, 1H), 8.26 (d, *J* = 8.6 Hz, 1H), 8.12 − 7.99 (m, 2H), 7.87 (dd, *J* = 1.8 Hz, *J* = 8.6 Hz, 1H), 7.56 − 7.44 (m, 2H), 3.52 (t, *J* = 5.5 Hz, 4H), 2.00 (m, 2H); ^13 ^C NMR (75 MHz, DMSO-d_6_) *δ* 164.8, 159.0, 155.6, 140.4, 139.3, 135.5, 134.9, 128.0, 127.1, 126.3, 125.8, 125.5, 125.3, 123.0, 122.9, 122.8, 38.9 (2 C), 17.7; Analysis calcd for C_19_H_16_ClN_3_S_2_: C, 59.13; H, 4.18; N, 10.89%; Found: C, 59.19; H, 4.06; N, 10.98%.

##### 2.1.2.6. 2-(1*H*-Indol-3-yl)-6–(3,4,5,6-tetrahydropyrimidin-1-ium-2-yl)benzothiazole chloride 5f

Compound **5f** was prepared from 1*H*-indole-3-carbaldehyde **4f** (0.073 g, 0.5 mmol). The resulting free base was converted into salt as described below to obtained 0.050 g (27.2%) of coloreless powder; m.p. >300 °C; ^1^H NMR (300 MHz, DMSO-*d*_6_) *δ* 12.25 (s, 1H), 10.23 (s, 2H), 8.51 (d, *J* = 1.7 Hz, 1H), 8.42 − 8.37 (m, 2H), 8.13 (d, *J* = 8.5 Hz, 1H), 7.83 (dd, *J* = 1.8 Hz, *J* = 8.6 Hz, 1H), 7.57 (m, 1H), 7.34 − 7.25 (m, 2H), 3.53 (t, *J* = 5.5 Hz, 4H), 2.02 (m, 2H); ^13 ^C NMR (75 MHz, DMSO-d_6_) *δ* 166.7, 158.8, 156.7, 136.9, 133.3, 130.3, 125.6, 124.4, 123.7, 123.0, 122.0, 121.5, 121.4, 120.6, 112.6, 110.1, 38.8 (2 C), 17.8; Analysis calcd for C_19_H_17_ClN_4_S: C, 61.86; H, 4.65; N, 15.19%; Found: C, 61.97; H, 4.69; N, 15.02%.

##### 2.1.2.7. 2-(Benzothiazole-2-yl)-6–(3,4,5,6-tetrahydropyrimidin-1-ium-2-yl)benzothiazole chloride 5 g

Compound **5 g** was prepared from Benzothiazole-2-carbaldehyde **4 g** (0.082 g, 0.5 mmol). The resulting free base was converted into salt as described below to obtained 0.069 g (35.8%) of white powder; m.p. >300 °C; ^1^H NMR (300 MHz, DMSO-*d*_6_) *δ* 10.28 (s, 2H), 8.71 (s, 1H), 8.42 (d, *J* = 8.6 Hz, 1H), 8.30 (d, *J* = 7.3 Hz, 1H), 8.23 (d, *J* = 7.6 Hz 1H), 7.94 (d, *J* = 9.2 Hz, 1H), 7.72 − 7.59 (m, 2H), 3.55 (m, 4H), 2.02 (m, 2H); ^13 ^C NMR (75 MHz, DMSO-d_6_) *δ* 158.8, 155.2, 152.7, 135.2, 135.0, 127.2, 127.1, 126.5, 126.2, 123.6, 123.0, 122.7, 38.7 (2 C), 17.5; Analysis calcd for C_18_H_15_ClN_4_S_2_: C, 55.88; H, 3.91; N, 14.48%; Found: C, 55.99; H, 3.78; N, 14.56%.

##### 2.1.2.8. 2-(Naphthalene-2-yl)-6–(3,4,5,6-tetrahydropyrimidin-1-ium-2-yl)benzothiazole chloride 5 h

Compound **5 h** was prepared from 2-naphthaldehyde **4 h** (0.078 g, 0.5 mmol). The resulting free base was converted into salt as described below to obtained 0.078 g (41.1%) of pale yellow powder; m.p. >300 °C; ^1^H NMR (300 MHz, DMSO-*d*_6_) *δ* 10.25 (s, 2H), 8.79 (s, 1H), 8.67 (s, 1H), 8.35 − 8.18 (m, 3H), 8.14 (d, *J* = 8.6 Hz, 1H), 8.05 (m, 1H), 7.90 (m, 1H), 7.72 − 7.62 (m, 2H), 3.55 (t, *J* = 4.8 Hz, 4H), 2.02 (m, 2H); ^13 ^C NMR (75 MHz, DMSO-d_6_) *δ* 158.6, 156.0, 134.6, 134.3, 132.5, 129.5, 128.9, 128.7, 127.9, 127.8, 127.5, 127.0, 125.8, 125.1, 123.6, 122.7, 122.5, 38.7 (2 C), 17.8; Analysis calcd for C_21_H_18_ClN_3_S: C, 66.39; H, 4.78; N, 11.06%; Found: C, 66.28; H, 4.90; N, 11.09%.

##### 2.1.2.9. 2-(Phenanthrene-9-yl)-6–(3,4,5,6-tetrahydropyrimidin-1-ium-2-yl)benzothiazole chloride 5i

Compound **5i** was prepared from phenanthrene-9-carbaldehyde **4i** (0.103 g, 0.5 mmol). The resulting free base was converted into salt as described below to obtained 0.097 g (45.1%) of colourless powder; m.p. 196–200 °C; ^1^H NMR (300 MHz, DMSO-*d*_6_) *δ* 10.34 (s, 2H), 9.05 − 8.98 (m, 2H), 8.95 (d, *J* = 8.3 Hz, 1H), 8.74 (d, *J* = 1.7 Hz, 1H), 8.54 (s, 1H), 8.42 (d, *J* = 8.6 Hz, 1H), 8.24 (d, *J* = 7.8 Hz, 1H), 7.97 (dd, *J* = 1.8 Hz, *J* = 8.6 Hz, 1H), 7.89 − 7.74 (m, 4H), 3.57 (m, 4H), 2.04 (m, 2H); ^13 ^C NMR (151 MHz, DMSO-d_6_) *δ* 170.9, 158.8, 156.1, 134.7, 131.9, 130.8, 130.3, 130.1, 129.7, 129.1, 128.2, 128.0, 127.8, 127.7, 127.6, 126.2, 126.0, 125.5, 123.5, 123.3, 123.0, 122.6, 38.8 (2 C), 17.7; Analysis calcd for C_25_H_20_ClN_3_S: C, 69.84; H, 4.69; N, 9.77%; Found: C, 69.84; H, 4.69; N, 9.77%.

#### 2.1.3. General method for preparation of compounds 6a–6g

Solution of equimolar amounts of aldehydes **4a**–**4i**, 2–(3,4-diaminophenyl)-3,4,5,6-tetrahydropyrimidin-1-ium chloride **3**, and sodium metabisulfite as oxidising reagents in dimethyl sulfoxide, was heated for 15 min at 160 °C. After the reaction mixture was cooled, water was added and reaction mixture was made alkaline with 20% NaOH. The resulting free base was filtered off, washed with water and dried. The free base was suspended in absolute ethanol and concd HCl was added. The reaction mixture was stirred at room temperature for 24 h and the resulting precipitate was filtered off.

##### 2.1.3.1. 2-Phenyl-5(6)–(3,4,5,6-tetrahydropyrimidin-1-ium-2-yl)benzimidazole chloride 6a

Compound **6a** was prepared from benzaldehyde **4a** (0.047 g, 0.44 mmol), 2–(3,4-diaminophenyl)-3,4,5,6-tetrahydropyrimidin-1-ium chloride **3** (0.100 g, 0.44 mmol), and sodium metabisulfite (0.084 g, 0.44 mmol) in dimethyl sulfoxide (0,8 ml). The resulting free base was converted into salt as described below to obtained 0.040 g (29.0%) of beige powder; m.p. >300 °C; ^1^H NMR (300 MHz, DMSO-*d*_6_) *δ* 10.22 (s, 2H), 8.47 − 8.45 (m, 2H), 8.19 (s, 1H), 7.94 (d, *J* = 8.4 Hz, 1H), 7.76 (d, *J* = 8.4 Hz, 1H), 7.69 (s, 3H), 3.53 (m, 4H), 2.02 (m, 2H); ^13 ^C NMR (75 MHz, DMSO-d_6_) *δ* 159.3, 152.7, 132.2, 129.3, 127.7, 123.8, 123.2, 115.0, 38.8, 17.7; Analysis calcd for C_17_H_17_ClN_4_: C, 65.28; H, 5.48; N, 17.91%; Found: C, 65.14; H, 5.61; N, 17.99%.

##### 2.1.3.2. 2–(4-Cyanophenyl)-5(6)–(3,4,5,6-tetrahydropyrimidin-1-ium-2-yl)benzimidazole chloride 6 b

Compound **6 b** was prepared from 4-cyanobenzaldehyde **4 b** (0.058 g, 0.44 mmol), 2–(3,4-diaminophenyl)-3,4,5,6-tetrahydropyrimidin-1-ium chloride **3** (0.100 g, 0.44 mmol), and sodium metabisulfite (0.084 g, 0.44 mmol) in dimethyl sulfoxide (1 ml). The resulting free base was converted into salt as described below to obtained 0.075 g (50.3%) of beige powder; m.p. >300 °C; ^1^H NMR (600 MHz, DMSO-d_6_) *δ* 14.20 (bs, 1H), 10.04 (s, 2H), 8.47 (d, *J* = 7.7 Hz, 2H), 8.11 (s, 1H), 8.06 (d, *J* = 7.8 Hz, 2H), 7.83 (d, *J* = 7.8 Hz, 1H), 7.62 (d, *J* = 8.0 Hz, 1H), 3.52 (m, 4H), 2.01 (m, 2H); ^13 ^C NMR (75 MHz, DMSO-d_6_) *δ* 160.1, 152.8, 134.1, 133.5, 128.0, 123.0, 122.4, 119.0, 113.0, 39.3, 18.4; Analysis calcd for C_18_H_16_ClN_5_: C, 64.00; H, 4.77; N, 20.73%; Found: C, 64.03; H, 4.74; N, 20.71%.

##### 2.1.3.3. 2–(4-Trifluoromethylphenyl)-5(6)–(3,4,5,6-tetrahydropyrimidin-1-ium-2-yl)benzimidazole chloride 6c

Compound **6c** was prepared from 4-trifluoromethylbenzaldehyde **4c** (0.077 g, 0.44 mmol), 2–(3,4-diaminophenyl)-3,4,5,6-tetrahydropyrimidin-1-ium chloride **3** (0.100 g, 0.44 mmol), and sodium metabisulfite (0.084 g, 0.44 mmol) in dimethyl sulfoxide (0.8 ml). The resulting free base was converted into salt as described below to obtained 0.101 g (60.1%) of beige powder; m.p. >300 °C; ^1^H NMR (300 MHz, DMSO-d_6_) *δ* 14.13 (bs, 1H), 10.05 (s, 2H), 8.51 (d, *J* = 8.1 Hz, 2H), 8.12 (s, 1H), 7.97 (d, *J* = 8.3 Hz, 2H), 7.84 (d, *J* = 8.1 Hz, 1H), 7.62 (d, *J* = 8.3 Hz, 1H), 3.53 (t, *J* = 5.5 Hz, 4H), 2.01 (m, 2H); ^13 ^C NMR (75 MHz, DMSO-d_6_) *δ* 160.1, 133.7, 131.0, 130.5, 128.1, 126.5, 126.5, 126.3, 123.0, 122.7, 39.3, 18.4; Analysis calcd for C_18_H_16_ClF_3_N_4_: C, 56.77; H, 4.24; N, 14.71%; Found: C, 56.89; H, 4.21; N, 14.60%.

##### 2.1.3.4. 2-(Benzo[*b*]thiophen-2-yl)-5(6)–(3,4,5,6-tetrahydropyrimidin-1-ium-2-yl)benzimidazole chloride 6d

Compound **6d** was prepared from benzo[*b*]thiophene-2-carbaldehyde **4e** (0.054 g, 0.33 mmol), 2–(3,4-diaminophenyl)-3,4,5,6-tetrahydropyrimidin-1-ium chloride **3** (0.075 g, 0.33 mmol), and sodium metabisulfite (0.063 g, 0.33 mmol) in dimethyl sulfoxide (0.6 ml). The resulting free base was converted into salt as described below to obtained 0.053 g (43.1%) of beige powder; m.p. >300 °C; ^1^H NMR (600 MHz, DMSO-*d*_6_) *δ* 10.16 (s, 2H), 8.23 (s, 2H), 8.00 (s, 1H), 8.00 (d, *J* = 6.6 Hz, 1H), 7.91 (d, *J* = 6.8 Hz, 1H), 7.65 (d, *J* = 8.3 Hz, 1H), 7.47 (d, *J* = 8.1 Hz, 1H), 7.43 − 7.36 (m, 2H), 3.51 (t, *J* = 5.3 Hz, 4H), 1.99 (m, 2H); ^13 ^C NMR (75 MHz, DMF-*d*_7_) *δ* 160.7, 140.8, 140.6, 126.0, 125.6, 125.0, 123.4, 120.5, 116.3, 39.8, 19.1; Analysis calcd for C_19_H_17_ClN_4_S: C, 61.86; H, 4.65; N, 15.19%; Found: C, 61.88; H, 4.62; N, 15.21%.

##### 2.1.3.5. 2-(1*H*-Indol-3-yl)-5(6)–(3,4,5,6-tetrahydropyrimidin-1-ium-2-yl)benzimidazole chloride 6e

Compound **6e** was prepared from 1*H*-indole-3-carbaldehyde **4f** (0.064 g, 0.44 mmol), 2–(3,4-diaminophenyl)-3,4,5,6-tetrahydropyrimidin-1-ium chloride **3** (0.100 g, 0.44 mmol), and sodium metabisulfite (0.084 g, 0.44 mmol) in dimethyl sulfoxide (0.8 ml). The resulting free base was converted into salt as described below to obtained 0.033 g (21.3%) of light brown powder; m.p. >300 °C; ^1^H NMR (600 MHz, DMSO-*d*_6_) *δ* 11.82 (s, 1H), 10.16 (s, 2H), 8.51 (d, *J* = 7.2 Hz, 1H), 8.35 (s, 1H), 7.97 (s, 1H), 7.70 (d, *J* = 8.1 Hz, 1H), 7.52 (d, *J* = 8.0 Hz, 2H), 7.24 (t, *J* = 6.8 Hz, 1H), 7.21 (t, *J* = 6.8 Hz, 1H), 3.52 (t, *J* = 5.5 Hz, 4H), 1.99 (m, 2H); ^13 ^C NMR (151 MHz, DMSO-d_6_) *δ* 159.5, 136.6, 127.4, 125.2, 122.3, 121.4, 121.3, 120.5, 120.5, 112.0, 105.9, 39.0, 18.7; Analysis calcd for C_19_H_18_ClN_5_: C, 64.86; H, 5.16; N, 19.91%; Found: C, 64.83; H, 5.17; N, 19.90%.

##### 2.1.3.6. 2-(Naphthalene-2-yl)-5(6)–(3,4,5,6-tetrahydropyrimidin-1-ium-2-yl)benzimidazole chloride 6f

Compound **6f** was prepared from 2-naphthaldehyde **4 h** (0.069 g, 0.44 mmol), 2–(3,4-diaminophenyl)-3,4,5,6-tetrahydropyrimidin-1-ium chloride **3** (0.100 g, 0.44 mmol), and sodium metabisulfite (0.084 g, 0.44 mmol) in dimethyl sulfoxide (0.8 ml). The resulting free base was converted into salt as described below to obtained 0.081 g (50.6%) of beige powder; m.p. >300 °C; ^1^H NMR (600 MHz, DMSO-d_6_) *δ* 10.25 (s, 2H), 8.80 (d, *J* = 7.2 Hz, 1H), 8.27 (s, 1H), 8.24 (d, *J* = 8.6 Hz, 1H), 8.17 (d, *J* = 7.2 Hz, 1H), 8.13 (d, *J* = 7.5 Hz, 1H), 7.97 (d, *J* = 8.4 Hz, 1H), 7.83 − 7.74 (m, 2H), 7.73 − 7.65 (m, 2H), 3.55 (m, 4H), 2.03 (m, 2H); ^13 ^C NMR (151 MHz, DMSO-*d*_6_) *δ* 159.4, 153.3, 133.4, 131.7, 130.1, 129.3, 128.6, 127.7, 126.7, 125.5, 125.3, 124.7, 123.2, 122.6, 115.5, 115.0, 38.9, 17.8

Analysis calcd for C_21_H_19_ClN_4_: C, 69.51; H, 5.28; N, 15.44%; Found: C, 69.49; H, 5.30; N, 15.48%.

##### 2.1.3.7. 2-(Phenanthrene-9-yl)-5(6)–(3,4,5,6-tetrahydropyrimidin-1-ium-2-yl)benzimidazole chloride 6 g

Compound **6 g** was prepared from phenanthrene-9-carbaldehyde **4i** (0.091 g, 0.44 mmol), 2–(3,4-diaminophenyl)-3,4,5,6-tetrahydropyrimidin-1-ium chloride **3** (0.100 g, 0.44 mmol), and sodium metabisulfite (0.084 g, 0.44 mmol) in dimethyl sulfoxide (0.8 ml). The resulting free base was converted into salt as described below to obtained 0.077 g (42.3%) of beige powder; m.p. >300 °C; ^1^H NMR (300 MHz, DMSO-*d*_6_) *δ* 10.27 (s, 2H), 9.02 (d, *J* = 8.1 Hz, 1H), 8.97 (d, *J* = 8.2 Hz, 1H), 8.88 (d, *J* = 8.0 Hz, 1H), 8.56 (s, 1H), 8.29 (s, 1H), 8.16 (d, *J* = 7.4 Hz, 1H), 7.97 (d, *J* = 8.5 Hz, 1H), 7.90 − 7.74 (m, 5H), 3.55 (m, 4H), 2.03 (m, 2H); ^13 ^C NMR (75 MHz, DMSO-d_6_) *δ* 159.8, 153.8, 131.5, 131.1, 130.7, 130.5, 130.0, 129.4, 129.0, 128.1, 128.0, 127.0, 124.0, 123.6, 123.5, 123.0, 39.3, 18.3; Analysis calcd for C_25_H_21_ClN_4_: C, 72.72; H, 5.13; N, 13.57%; Found: C, 72.76; H, 5.10; N, 13.52%.

### 2.2. Biology

#### 2.2.1. Antitumor activity in 2 D and 3 D assays

##### 2.2.1.1. Material and methods

###### 2.2.1.1.1. Test compounds

Doxorubicin was purchased from Apollo (BID0120; Opelika, AL), staurosporine was purchased from Biotrend (BS0188; Zurich, SUI) and vandetanib was purchased from Selleckchem (S1046, Houston, TX, USA). Test compounds were synthesised by research group at Department of Organic Chemistry, Faculty of Chemical Engineering and Technology, University of Zagreb.

Mother plates (96-well-V plates, polypropylene, Greiner Bio-one, Cat. 651201) with serial dilutions of compounds in pure DMSO are prepared from 10 mM DMSO stock solutions on Janus automatic pipetting workstation (Perkin-Elmer). Compounds are diluted 1:3. 500 nL (for 2 D cell culture) or 400 nL (for 3 D cell culture) of compound were transferred from mother plate to test plate by using Mosquito (TTP labtech). DMSO percentage in test concentrations was 0.5 − 1.0%. Starting concentration of the test compounds and standard compound doxorubicin was 50 µM (2 D cell culture) or 100 µM (3 D cell culture).

Starting concentration of staurosporine was 10 µM in both, 2 D and 3 D cell culture assays, while starting concentration of vandetanib was 25 µM (2 D cell culture) or 50 µM (3 D cell culture).

###### 2.2.1.1.2. Cell cultures

Human lung cancer cell lines A549 (ATCC CCL-185), HCC827 (ATCC CRL-2868) and NCI-H358 (ATCC CRL-5807) were purchased from American Type Culture Collection (ATCC, Manassas, VA, USA).

Cells were maintained in appropriated, recommended by supplier medium, supplemented with 10% heat inactivated foetal bovine serum and penicillin/streptomycin, in incubator (INCO2, Memmert) in a humidified atmosphere of 5% CO_2_ and 95% O_2_ at 37 °C.

##### 2.2.1.2. High-throughput 2 D and 3 D drug screening setup

###### 2.2.2.2.1. 2 D cell culture assay

Cells were grown in 96 well cell star polystyrene plates. 10,000 cells/well were seeded in wells 4 h prior the treatment with different compounds concentrations and incubated for 72 h. A cell viability assay was performed according to the manufacturer’s instruction, using CellTiter 96 aqueous solution, MTS kit (Promega, Madison, WI, USA). After 0.5–2 h of cell incubation with MTS in 2 D cell culture, the plates were read using PE EnVision absorbance at 490 nm. The results for each of tested compounds are reported as growth percentages from two independent concentrations curves compared with the untreated control cells after drug exposure. Since we prefer homogeneous assays (mix and read procedures) and therefore MTS is more practical (one step less than MTT). MTS is more sensitive and accurate in comparison with MTT assay.

###### 2.2.2.2.2. 3 D cell culture assay

Cells were grown in 96 well Perfecta 3 D hanging drop plates 5000 cell/well for 4 days until spheres were formed (and checked under the microscope). After sphere formation cells were treated with compounds followed by 72 h incubation. Cell viability assay was performed according to the manufacturer’s instruction, using CellTiter GLO 3 D for 3 D cell culture (Promega); cell incubation was 5 min on the shaker followed by 25 min incubation in the dark. Plates were read using PE EnVision luminescence. The results for each of tested compounds are reported as growth percentages from two independent concentrations curves compared with the untreated control cells after drug exposure.

##### 2.2.1.3. BrdU proliferation assay

Proliferation assay was performed using Cell Proliferation ELISA, BrdU (Sigma, St. Louis, MO, USA). Briefly, 48 h hours after compound addition, 10 µM of BrdU was added to each well and incubated for following 24 h. After incubation, single cell suspension was made for 3 D cell culture in the new 96 well plate. Plates were centrifuged and supernatants were removed from each plate (for 2 D and 3 D cell culture) followed by 60 min incubation at 60° C. The rest of the proliferation assay was performed according to the manufacturer instructions.

##### 2.2.1.4. Annexin V assay – apoptotic changes in plasma membrane

Under physiological conditions, choline phospholipids (phosphatidylcholine, sphingomyelin) are exposed on the external leaflet while aminophospholipids (phosphatidylserine, phosphatidylethanolamine) are exclusively located on the cytoplasmic surface of the lipid bilayer. This asymmetry is scrambled during apoptosis when phosphatidylserine becomes exposed on the extracellular side of the membrane^33^.

Phosphatidylserine is detected by anticoagulant protein Annexin V (tagged with fluorochrome) that reversibly binds to phosphatidylserine residues on the extracellular side of the membrane. Annexin V apoptotic assay was performed using Annexin V Alexa Fluor 488 conjugate (Thermo Fisher Scientific, Waltham, MA, USA) according to manufacturer instruction. Briefly, 1 × 10^5^ cells were seeded on 24 well plate and treated with IC_50_ concentration of chosen compounds from amidine series for 36 h. Cells suspensions were collected after incubation into 5 ml Falcon tubes, washed and centrifuged 2 times (once with PBS, followed by Annexin V Binding Buffer (AVBB)) for 5 min, 400 g at room temperature (RT). 100 µL of AVBB was added to each tube followed by addition of 2 µL of Annexin V Alexa Fluor 488 conjugate and incubated for 15 min at RT. Cells were washed in AVBB for 5 min, 400 g at RT. SYTOX™ AADvanced™ Dead Cell Stain Kit (Thermo Fisher Scientific) was used for detection of late apoptotic and necrotic cells. 1 µL of SYTOX AADvanced was added to each tube and incubated at RT for 5 min in dark. 500 µL of AVBB was added and samples were kept on ice until analysed. The results for each of tested compounds are reported as percentage of positive cells (live cells (Annexin V−/SytoxAAD−), apoptotic cells (Annexin V+/SytoxAAD−), late apoptotic/necrotic cells (Annexin V+/SytoxAAD+) and necrotic cells (Annexin V−/SytoxAAD+)).

##### 2.2.1.5. Statistical analysis

Calculation of IC_50_ data, curves and QC analysis is made by using Excel tools and GraphPadPrism software (La Jolla, CA), v. 5.03. In brief, individual concentration–effect curves are generated by plotting the logarithm of the tested concentration of tested compounds (X) versus corresponding percent inhibition values (Y) using least squares (ordinary) fit. Best fit IC_50_ values are calculated using Log(inhibitor) versus normalised response – Variable slope equation, where Y = 100/(1 + 10((LogIC_50_ – X) * HillSlope)). QC criteria parameters (Z', S:B, R2, HillSlope) were checked for every IC_50_ curve.

#### 2.2.2. Dmpk *in vitro* analyis

##### 2.2.2.1. Materials

Dimethyl sulfoxide (DMSO), phosphate-buffered saline (PBS), nicotinamide adenine dinucleotide phosphate (NADP), glucose-6-phosphate, glucose-6-phosphate dehydrogenase, magnesium chloride, Dulbecco’s phosphate-buffered saline (D-PBS), Dulbecco’s Modified Eagles medium (DMEM), fetalbovine serum (FBS), glutamax, nonessential amino acids (NEAA), EDTA, 0.05% Trypsin-EDTA, ammonium acetate, sulfaphenazole, α–naphtoflavone, propranolol, caffeine, acebutolol, verapamil, nicardipine warfarin sodium, benfluorex hydrochloride, eucatropine hydrochloride and diclofenac were purchased from Sigma Aldrich (St. Louis, MO, USA). Ammonia solution, min. 25% p.a. was obtained from Kemika (Zagreb, Croatia). Lucifer yellow was purchased from EndoTherm (Saarland University, Germany), elacridar from International Laboratory (South San Francisco, CA, USA) and amprenavir form AKSci (Union City, CA, USA). Acetonitrile, methanol, sodium hydrogen phosphate, potassium dihydrogen phosphate ethanol and sodium chloride were obtained from Merck (Darmstadt, Germany) and acetonitrile ultra gradient grade HPLC analysed from J.T. Baker, Fisher Scientific (Pittsburgh, PA, USA). BCA Protein Assay Kit was purchased from Thermo Scientific (Waltham, MA, USA), 4-androsten-17b-ol-3-one (testosterone) from Steraloids (Newport, RI, USA) and human and mouse liver microsomes from Corning (New York, NY, USA). MDCKII-hMDR1 cells were obtained from Solvo Biotechnology (Szeged, Hungary), antibiotic/antimycotic from Gibco, Thermo Scientific (Waltham, MA, USA) and physiological solution, 0.9% NaCl from Croatian Institute for Transfusion Medicine (Zagreb, Croatia). Human and mouse plasma were purchased from BioIVT/Seralab (Sussex, United Kingdom).

##### 2.2.2.2. Kinetic solubility

Test compounds and assay controls (sulfaphenazole and α-naphtoflavone) were serially diluted in DMSO by factor 3.3x, 3x, 3.3x and 3x, resulting in a total of 5 different concentrations. Phosphate buffer (50 mM, pH 7.4) was spiked with diluted test compounds and controls with final concentration range in the assay as follows: 100, 30, 10, 3 and 1 µM (1% DMSO). Plate was then incubated by gentle shaking (200–300 rpm) for 1 h and 45 min at 37 °C. After additional15 min on room temperature (without shaking), plate absorbance at 620 nm was measured with microplate reader Tecan, Infinite F500 (total incubation time = 2 h).

##### 2.2.2.3. Chromatographic lipophilicity study

In order to determine the lipophilicity range, Chromatographic Hydrophobicity Index was measured by gradient reverse-phase HPLC at physiological pH. Sample working solutions were prepared from 10 mM DMSO stock solutions by dilution with acetonitrile to a final concentration of 1.25 mM and analysed on Agilent 1100 Series liquid chromatography system with HPLC diode-array detector (DAD) coupled with Micromass Quattro micro API mass spectrometer. Samples were injected onto an HPLC column (Phenomenex Luna C18, 50 × 3 mm, 5 µm) and eluted with a gradient at room temperature (sample temperature 15 °C). The mobile phase was composed of 50 mM Ammonium acetate, pH = 7.4 and acetonitrille. A total run time was 5 min, with the flow rate of 1 ml/min (under gradient conditions). The HPLC system was initially calibrated using the calibration set of 10 compounds with literature CHI values. The experimentally determined gradient retention times were plotted against the CHI_7.4_ values published in the literature resulting in the equation obtained from linear regression analysis. This equation was further used for the calculation of CHI_7.4_ for test compounds from determined retention times of the main chromatographic peak from UV chromatogram for each compound. The obtained CHI_7.4_ values were further converted to Chrom log D_7.4_ as follows: CHI_7.4_ × 0.0857 − 2.

##### 2.2.2.4. Metabolic stability in liver microsomes

Tested compounds (final concentration of 1 µM, 0.1% DMSO), as well as testosterone and propranolol as positive controls and caffeine as negative control were incubated in phosphate buffer (50 mM, pH 7.4) for 60 min at 37 °C with liver microsomes (human and mouse) in the absence and presence of the NADPH cofactor. The NADPH generating system was prepared in phosphate buffer and consisted of nicotinamide adenine dinucleotide phosphate (NADP, 0.38 mM), glucose-6-phosphate (1.49 mM), glucose-6-phosphate dehydrogenase (1.5 U) and magnesium chloride (0.1 mM). Aliquots were taken at different time points (0, 10, 20, 30, 45 and 60 min) and reaction was terminated by addition of a MeCN/MeOH (2:1) mixture, containing diclofenac as internal standard. Samples were then centrifuged (at 4500 rpm, at 4 °C, for 30 min) and resulting supernatants were subjected to LC-MS/MS analysis. The *in vitro* half life (t_½_) was determined from the slope of the linear regression of ln % parent compound remaining versus incubation time. *In vitro* intrinsic clearance, expressed as µl/min/mg liver, was determined from *in vitro* half life (t_½_) and normalised for the protein concentration in the incubation mixture. Predicted *in vivo* hepatic clearance was determined from *in vitro* intrinsic clearance assuming 52.5 mg of protein/g of liver and using constant values for liver weight/body weight [g/kg] (25.7 for human, 87.5 for mouse) and liver blood flow (LBF) [ml/min/kg] (21 for human and 131 for mouse).

##### 2.2.2.5. Plasma protein binding

The extent of binding to plasma proteins was assessed using equilibrium dialysis method. Plasma (human and mouse) was spiked with test compounds and three controls (nicardipine and verapamil in both species, acebutolol in human and caffeine in mouse plasma) to obtain final concentration of 5 µM (0.5%DMSO). Hydrated membranes were inserted into equilibrium dialysis unit (HT Dialysis) according to manufacturer’s instructions. The dialysate side was then loaded with appropriate volume of buffer, while the same volume of spiked plasma was added into sample side of the well. Incubation lasted 4 h at 37 °C with gentle shaking. Afterwards, protein precipitation was done by mixing matrix matched aliquot of plasma or buffer with 3 volumes of MeCN/MeOH (2:1) mixture, containing internal standard (diclofenac). After centrifugation (at 4500 rpm, at 4 °C, for 30 min) resulting supernatants were subjected to LC-MS/MS.

##### 2.2.2.7. Stability in mouse and human plasma

Tested compounds (final concentration of 5 µM, 0.5% DMSO) were incubated for 4 h at 37 °C in human and mouse plasma, respectively. Aliquots were taken at different time points (0, 30, 120 and 240 min) and reaction was terminated by addition of a MeCN/MeOH (2:1) mixture, containing internal standard (diclofenac). Samples were then centrifuged (at 4500 rpm, at 4 °C, for 30 min) and resulting supernatants were subjected to LC-MS/MS analysis. The same procedure was followed for controls used in the assay: propranolol (both species), benfluorex (mouse) and eucatropine (human), respectively.

##### 2.2.2.8. MDCKII-MDR1 permeability assay

Permeability and P-glycoprotein substrate assessment was done on MDCKII-hMDR1, Madin-Darby canine epithelial cells over-expressing human MDR1 gene, coding for P-glycoprotein.

Cells were prepared for transport studies by seeding on 96-well cell culture inserts (Millipore, MA, USA) in a concentration of 0.25 × 10^6^ cells per ml. The cells were fed with fresh medium 24 h post seeding and cultured to confluence for 3 days before use. On the day of experiment, the cell monolayers were washed and equilibrated with transport medium (DPBS, pH 7.4 containing 1% DMSO) with or without P-gp specific inhibitor, elacridar (2 µM) for 45 min (37 °C, 5% CO_2_, 95% humidity). Test compound solution consisted of test substance (10 μM) in D-PBS medium containing lucifer yellow (100 μM) and 1% DMSO. Transport assays were conducted in apical to basolateral (A2B) and basolateral to apical (B2A) directions, respectively. Monolayers were incubated with the compound solution for 60 min at 37 °C under gentle agitation. Apical and basolateral compartments were sampled at the end of the transport experiment, while donor solutions were also sampled at the beginning of the experiment in order to determine initial concentration. Test substance concentrations in both compartments were determined by LC-MS/MS. There were several controls used in the assay: 1) amprenavir (0.5 μM) served as a low permeable control, being also a P-gp substrate; 2) diclofenac (10 μM) was used as a high permeable control; 3) Lucifer yellow, a fluorescent marker for the paracellular membrane transport, was used as a control of cell monolayer integrity.

##### 2.2.2.9. LC-MS/MS analysis

All ADME samples were analysed on a Sciex API 4000 or Sciex API4500 Triple Quadrupole Mass Spectrometer (Sciex, Framingham, MA, USA) coupled to a UHPLC System (Shimadzu Nexera X2; Shimadzu, Kyoto, Japan). Samples were injected onto an UPLC column (HALO2 C18, 2.1 × 20 mm, 2 µm or Phenomenex Luna Omega Polar C18, 30 × 2.1 mm, 1.6 µm) and eluted with a gradient at temperature of 50 °C. The mobile phase was composed of acetonitrile (with 0.1% formic acid) and 0.1% formic acid in deionised water. A total run time was 1 or 1.5 min, with the flow rate of 0.7 ml/min (under gradient conditions). A positive ion mode with turbo spray, an ion source temperature of 500 °C and a dwell time of 150 ms were utilised for mass spectrometric detection. Multiple reaction monitoring (MRM) was used at the specific transitions for each compound/control tested: compound **5c**: 361.9 → 304.9; compound **5d**: 344.8 → 287.9; compound **5e**: 349.8 → 292.9; compound **5 g**: 350.8 → 293.9; compound **5 h**: 344.0 → 286.9; compound **6a**: 276.9 → 220.0; compound **5 b**: 319.0 → 262.0; compound **6 b**: 302.1 → 245.1; compound **6c**: 344.9 → 288.0; compound **6d**: 333.0 → 276.0; compound **6e**: 316.1 → 259.0; compound **6f**: 327.1 → 270.0; compound **6 g**: 377.1 → 320.1; testosterone: 289.3→ 97.1; propranolol: 260.1 → 182.8; caffeine: 195.2 → 138.1; acebutolol: 337.2 → 116.2, nicardipine: 480.2 → 315.0; verapamil: 455.4 → 165.1; amprenavir: 506.2 → 245.4; diclofenac: 296.1 → 213.7 or 296.2 → 215.3; eucatropine: 292.2 → 109.1; benfluorex: 352.1 → 230.3, and warfarin: 309.2 → 163.2

## 3. Results and discussion

### 3.1. Chemistry

The targeted amidino substituted benzothiazoles **5a–5i** and benzimidazoles **6a–6g** were synthesised according to the procedure shown in Scheme 1 by using conventional methods for cyclocondenastion to fused benzazole derivatives. Within the cyclocondensation in refluxing acetic acid between commercially aryl aldehydes **4a–4i** and amidino substituted benzenethiolate **2**, followed by quenching with hydrochloric acid, benzothiazoles **5a–5i** as hydrochloride salts obtained in moderate to good reaction yields. This method has been optimised in order to perform direct condensation of aldehydes with amidino substituted 2-aminothiophenoles without using any catalyst or oxidant. The precursor 2-amino-5–(3,4,5,6-tetrahydropyrimidin-1-ium-2-yl)benzenethiolate **2** in the form of zwitterion was prepared from 6-cyanobenzothiazole by Pinner reaction according to our previously described and well developed method^34^.

Amidino substituted benzimidazole derivatives **6a–6g** (Scheme 1) were prepared following the experimental protocol shown in the Scheme 1. Within the reaction of cyclocondensation, from substituted aryl aldehydes **4a–4i** and 2–(3,4-diaminophenyl)-3,4,5,6-tetrahydropyrimidin-1-ium chloride **3**, by using sodium metabisulfite as oxidising reagents, corresponding 2-aryl substituted benzimidazoles **6a–6g** as hydrochloride salts were prepared in moderate reaction yields. Cyclocondesation with 2-quinolinyl-carboxaldehyde **4d** and 2-benzothiazolylcarboxaldehyde **4 g** failed and desired product was not isolated successfully. Amidino substituted intermediar **3** obtained in the acidic Pinner reaction from corresponding cyano substituted precursors according to the previously published procedures^14^.

The structures of all newly prepared amidino substituted benzimidazole/benzothiazole derivatives were confirmed by means of ^1^H and ^13 ^C NMR spectroscopy. NMR analysis based on the values of chemical shifts and H–H coupling constants in the ^1^H spectra confirmed the structures of compounds. Furthermore, ^13 ^C NMR chemical shifts were consistent with the suggested structures. Also, IR spectroscopy was used for the monitoring of Pinner reaction due to the synthesis of main precursors **2** and **3.**

### 3.2. Antiproliferative activity *in vitro*

We have tested compounds in MTS cytotoxicity assay and in BrdU proliferative assay. MTS assay give us information of living cells number, in this case, metabolically active cells, whereas BrdU proliferation assay give as a measure of cell population in active division phase.

To avoid that some prominent compounds can be discarded in early screening phase and to give a chance to further profiling only to compounds active on 2 D classical format assays, we tested compounds in both format assays 2 D and 3 D. Both assays were performed on 2 D and 3 D assay format. For 2 D cell assay format, we used a classic two-dimensional *in vitro* assay and as 3 D assay, we used a hanging drop proliferation cell assay previously described.

We have tested the antiproliferative activity on 2 D and 3 D cell culture assays *in vitro* of newly synthesised amidino substituted benzimidazole/benzothiazole derivatives **5a–5i** and **6a–6g** on three human lung cancer cell lines A549, HCC827 and NCI-H358. As standard drugs, *doxorubicin*, *staurosporine* and *vandetanib* were used. Doxorubicin interact with DNA by intercalation and inhibits synthesis of biomoleculas, staurosporin is protein kinase C (PKC) inhibitor and vandetanib multikinase inhibitor (VGFRs, EGFR and RET kinase) is antitumor drug with potential use in a broad range of tumours types, especially thyroid and lung. For 2 D cell assay we used a classic two-dimensional *in vitro* cancer cell line proliferation assay and as 3 D assay we used a hanging drop proliferation cell assay. Antiproliferative activity for each compound is presented as an IC_50_ value that was calculated using the program GraphPadPrism software (La Jolla, CA), v. 5.03., and average values from three independent experiments. The results for each of tested compounds are reported as growth percentages from two independent concentrations curves compared with the untreated control cells after drug exposure. In both assay formats, inhibition of proliferation was measured by MTS viability assay. Obtained IC_50_ inhibitory concentrations in 2 D and 3 D cell culture system for three human lung cancer cell lines are depicted in Table 1.

**Table 1.** Antitumor activity of prepared compounds in 2 D and 3 D cell cultures.

Compound	IC_50_ (µM)±SD; N = 2					
	*A549*		*HCC827*		*NCI-H358*	
	2D	3D	2D	3D	2D	3D
**5a**	>50	>100	>50	>100	>50	>100
**5b**	>50	60 ± 1.44	>50	>100	>50	>100
**5c**	34 ± 8.65	16 ± 0.65	7 ± 0.45	12 ± 0.16	23 ± 0.79	34 ± 0.70
**5d**	>50	16 ± 1.3	14 ± 2.09	>100	20 ± 2.53	40 ± 4.89
**5e**	36 ± 6.37	23 ± 6.24	7 ± 0.12	22 ± 9.45	16 ± 0.98	34 ± 0.16
**5f**	>50	>100	>50	>100	>50	>100
**5g**	41 ± 0.54	15 ± 1.35	19 ± 2.21	17 ± 0.93	26 ± 0.30	31 ± 0.05
**5h**	38 ± 3.68	14 ± 0.38	6 ± 0.05	12 ± 0.26	10 ± 1.00	31 ± 0.33
**5i**	>50	>100	>50	>100	>50	>100
**6a**	15 ± 1.70	13 ± 1.46	6 ± 0.5	9 ± 3.05	13 ± 0.28	17 ± 1.94
**6b**	>50	>100	>50	>100	>50	>100
**6c**	>50	>100	>50	>100	>50	>100
**6d**	>50	>100	>50	>100	>50	>100
**6e**	>50	>100	>50	>100	>50	>100
**6f**	>50	>100	>50	>100	>50	>100
**6g**	>50	>100	>50	>100	>50	>100
**Doxorubicin**	2 ± 0.23	5 ± 0.26	0.39 ± 0.05	0.78 ± 0.08	0.11 ± 0.01	0.25 ± 0.03
**Staurosporine**	1 ± 0.07	0.16	0.15 ± 0.01	0.02 ± 0.00	0.15 ± 0.02	0.12 ± 0.0
**Vandetanib**	>25	>50	0.81 ± 0.05	2 ± 0.11	3 ± 0.85	0.30 ± 0.02

As presented in Table 1, several compounds, mosty benzothiazole derivatives (**5c–e, 5 g, 5 h**) and one benzimidazole derivative **6a**, displayed antitumor activity in 2 D as well as in 3 D assays against all three cancer cells. Benzothiazole derivatives **5c** and **5d** show same or slightly lower activity in 2 D MTS assay format in comparison to 3 D format. Benzothiazole derivatives **5e**, **5 g**, **5 h** activity was also same or lower in 2 D format in comparison with IC values on 3 D assay format with exception for A549 cell line (Tabel 1) where copmpounds showed higher activity in 3 D format. In proliferative (BrdU) assay, activity of benzothiazole derivatives **5d**, **5e**, **5 g** were same or lower on 2 D format in comparison to 3 D (Table 2) while **5c** and **5 h** showed same activity pattern prevous compounds with exception for A549 and NCI-H358 cell lines where activity were opposite: higher on 3 D assay format. The most potent compound was benzimidazole derivative **6a** substituted with phenyl ring at position 2 with the lowest IC_50_ values on both assays, MTS and BrdU, and in format assay, 2 D and 3 D.

**Table 2.** Proliferation activity of prepared compounds in 2 D and 3 D cell cultures.

Compound	IC_50_ (µM)±SD; N = 2					
	*A549*		*HCC827*		*NCI-H358*	
	2D	3D	2D	3D	2D	3D
**5a**	>50	>100	>50	>100	>50	>100
**5b**	>50	87 ± 0.11	>50	>100	>50	>100
**5c**	48 ± 0.73	13 ± 0.78	15 ± 2.18	21 ± 1.41	16 ± 4.14	4.31
**5d**	1	16 ± 1.30	11 ± 0.17	32 ± 11.68	9 ± 0.31	13 ± 1.65
**5e**	16 ± 0.39	23 ± 6.24	10 ± 1.21	24 ± 7.63	9 ± 0.65	5 ± 0.81
**5f**	>50	>100	>50	>100	>50	>100
**5g**	7 ± 2.31	14 ± 3.07	18 ± 0.76	17 ± 0.93	13 ± 0.45	7 ± 2.33
**5h**	10 ± 0.78	14 ± 0.06	9 ± 0.83	12 ± 0.26	7 ± 0.07	1 ± 0.81
**5i**	>50	>100	>50	>100	>50	>100
**6a**	11 ± 5.56	12 ± 0.28	7 ± 0.35	12 ± 0.25	7 ± 0.3	3 ± 1.41
**6b**	>50	>100	>50	>100	>50	>100
**6c**	>50	>100	>50	>100	>50	>100
**6d**	>50	>100	>50	>100	>50	>100
**6e**	>50	>100	>50	>100	>50	>100
**6f**	>50	>100	>50	>100	>50	>100
**6g**	>50	>100	>50	>100	>50	>100
**Doxorubicin**	0.06 ± 0.01	0.39 ± 0.02	0.02 ± 0.00	1.03 ± 0.71	0.04 ± 0.00	0.34 ± 0.36
**Staurosporine**	0.23 ± 0.05	0.16 ± 0.06	0.06 ± 0.01	0.04 ± 0.01	0.15 ± 0.02	0.05 ± 0.04
**Vandetanib**	>25	1.25 ± 2.82	2.82 ± 1.98	0.87 ± 1.15	1 ± 0.28	1.91 ± 0.09

Other tested compounds showed very low activity or were not active at all. As we mention before, viability of cell (MTS assay) is a measure of the living cells whereas proliferation (BrdU) test is a measure of cell division (or proliferation rate). All active compounds (**5c–e, 5 g, 5 h and 6a**) showed strong activity in BrdU assay on NCI-H358 cell line, in comparison with MTS assay for the same cell line, meaning that compounds have promising antiproliferative effect with lower cytotoxicity potency. As we describe previoslly, cell grown in a 3 D environment support their natural 3 D physical shape and viable cells in the proliferating stage are mainly on the outer layer due to higher exposition to the medium^35^. We assume that low IC_50_ values in 3 D format assays for some active compounds are consequence of more exposed outer layer of proliferative cells on spheroids. Nevertheless, the proliferating rates cells in sferoids is depend on cell types, number of cells in inoculum, conditions in which cells are cultured but above of all is depend of cell line sensitivity and suscetability to antitumor drugs.

It is know that A49 cell line is less susceptible in comparison to other two cell lines and therefore IC_50_s values are higher. When compared to standard drugs, all derivatives were significantly less active except several compounds (**5c–e, 5 g, 5 h** and **6a**) which were more active against A549 cell line in comparison to *vandetanib*. Reagarding the obtained results of proliferation activity (Table 2), the smiliar results are obtained with the compounds **5c–e, 5 g, 5 h** and **6a** being the most active ones.

### 3.3. Annexin V assay – apoptotic changes in plasma membrane

**Figure 1.**
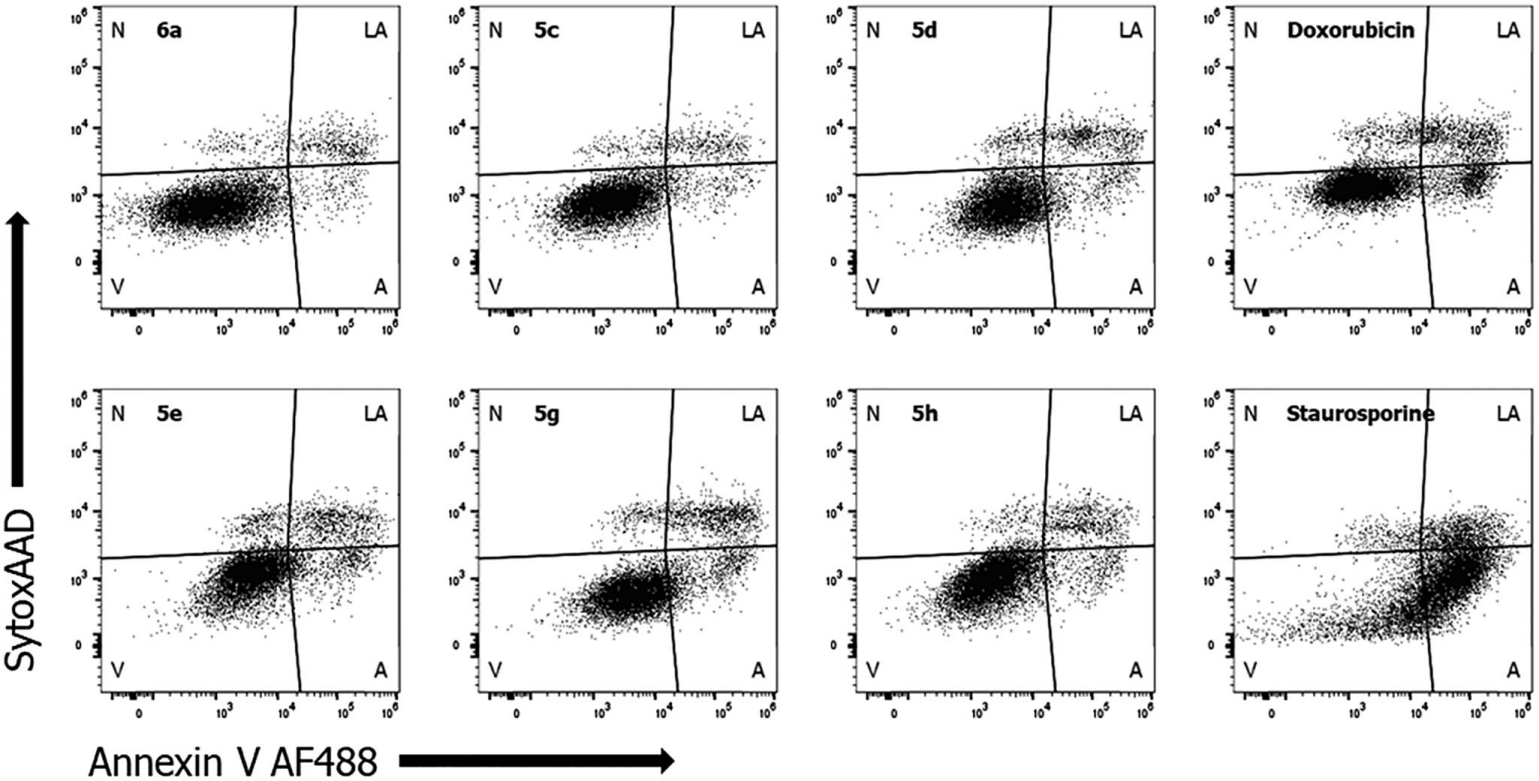
Annexin V staining to measure apoptosis. A549 cells were treated with active compounds (**6a**, **5c**–**e**, **5 g** and **5 h**) or standard compounds (doxorubicin and staurosporine) at determined IC_50_ value for 36 h to induce apoptosis in 2 D cell culture (V: viable cells; A: apoptotic; LA: late apoptotic; N: necrotic).

Mechanism of action of most active compounds in antiproliferative assay, benzothiazole derivatives **5c**–**e**, **5 g**, **5 h** and one benzimidazole derivative **6a**, was tested. As shown on Figure 1, all active compounds have similar mode of action on A549 cell line as standard compound doxorubicin, which is a cytotoxic anthracycline antibiotic that binds to nucleic acids by specific intercalation of the planar anthracycline nucleus with the DNA double helix. These results are in line with proposed chemical structure of novel amidino substituted benzimidazole/benzothiazole derivatives that suggest possible intercalation to DNA because of the structural similarity of benzimidazole scaffold with naturally occurring purines. Similar results were obtained for other cell lines tested (not shown).

### 3.4. Dmpk *in vitro* analysis

ADME results are presented in Table 3 (active compounds) and Table 4 (non-active compounds). To avoid mistake in our conclusion, that some compounds are active in 3 D because such cell cultures are more similar to *in vivo* conditions and that this is advantage in implementation of 3 D assay, we should excluded possibility that active compounds are active due to better ADME properties (especially solubility, and lipophilicity) in comparison to non-active compounds. Therefore, we profiled active and non-active compounds and see that ADME properties are the same for all tested compounds. In general, no significant difference was observed in ADME profile for these two set of compounds.

**Table 4.** Summary of ADME properties of non-nactive compounds.

	5b	6b	6c	6d	6e	6f	6g
Kinetic solubility range afer 2 h (µM)	>100	>100	>100	10–30	10–30	30–100	30–100
Chrom logD	2.18	1.17	2.60	2.43	1.38	2.18	3.27
Microsomes (1 µM) Predicted in vivo hep CL (% LBF)							
Mouse	<30	<30	<30	71	78	77	31
Human	<30	<30	<30	<30	<30	<30	<30
PPB % bound (recovery)							
Mouse	75.1 (98)	63.5 (87)	87.0 (117)	93.9 (89)	74.1 (91)	88.0 (102)	99.7 (95)
Human	66.6 (93)	54.3 (91)	85.6 (97)	92.6 (86)	65.1 (81)	81.0 (91)	98.5 (96)
Plasma stability (% remaining at 4 h)							
Mouse	95	119	94	92	84	NA	81
Human	86	98	96	95	98	106	85
MDCKII-MDR1							
Papp(A2B)	0.59	0.92	0.69	0.11	0.57	0.78	0.02
Papp(B2A)	20.5	1.02	1.93	0.86	2.17	2.49	0.2
Efflux ratio	35.4	1.13	2.82	7.75	3.89	3.23	6.6

#### 3.4.1. Kinetic solubility

Majority of compounds showed good (>100 µM) or moderate (30–100 µM) solubility after 2 h incubation with exception of low soluble (10–30 µM) compounds **5 h**, **6d** and **6e**.

#### 3.4.2. Chromatographic lipophilicity study

Majority of both active and inactive compounds have chromlogD_7.4_ values in the range 1–3.3 and in general, higher values are obtained for a set of active compounds. Exceptions to this are active compound **6a** with chromlogD_7.4_ value of 0.92, and inactive compound **6 g** that seems to be the one of the most lipophilic compounds with chromlogD_7.4_ value 3.27.

#### 3.4.3. Metabolic stability in liver microsomes

The *in vitro* metabolic stability, expressed as predicted *in vivo* hepatic clearance (% of liver blood flow, LBF), of selected compounds was investigated in human and mouse liver microsomes. Following incubation in liver microsomes majority of compounds are classified as stable molecules in both species and have low predicted *in vivo* clearance values (<30% LBF). As exceptions to this, compounds **5e** and **6 g** (in mouse liver microsomes) and **6a** (in both species) are characterised by moderate clearance (30–70% LBF). Compounds **6d**, **6e** and **6f** are characterised by high clearance (>70% LBF) in mouse liver microsomes.

#### 3.4.4. Plasma protein binding and stability in human and mouse plasma

Test compounds showed different range of binding to proteins of human and mouse plasma. In general, lower binding is observed in the group of inactive compounds, what could probably be explained by their lower lipophilicity (with exception of **6 g**). **6a**, **6 b**, **5 b** and **6e** are characterised by low binding in both species, with fraction bound (% Fb) between 54.3 and 75.1%. Compounds **6c**, **6d** and **6f**, are characterised by moderate binding in both species, with fraction bound (% Fb) between 80 and 95%. **5d** showed moderate binding in human plasma, with fraction bound of 94.7% and high binding in mouse plasma (% Fb= 96.0). On contrary, remaining compounds **5c**, **5e**, **5 g** and **5 h** in both species and **6 g** in human plasma are characterised by high binding, with fraction bound between 95 and 99%. In addition, **6 g**, one of the most lipophilic compounds, shows even higher binding, i.e. very high binding in mouse plasma, with fraction bound of 99.7%. All test compounds are stable in both human and mouse plasma (>70% after 4 h incubation). These results are in accordance with recovery values obtained in PPB experiment.

#### 3.4.5. Mdckii-MDR1 permeability assay

Both active and non-active compounds display low permeability, with P_app_(A2B) values below 2 × 10^−6 ^cm/sec in A2B direction without P-gp inhibitor (Tables 3 and 4). In the presence of elacridar (tested only for active compounds), A2B peremability, i.e. passive permeability, becomes moderate for compounds **5c**–**e** and **5 g**. For two remaining compounds, **5 h** and **6a**, permeability remains low (<2 × 10^−6 ^cm/sec) even in the presence of P-gp inhibitor. In addition, all active compounds could be classified as P-gp substrates with efflux ratio >2 in the absence of P-gp inhibitor and its significant decrease (at least 50%) in the presence of elacridar. Non-active compounds could also be classified as P-gp substrates based on efflux ratio (>2) in the absence of inhibitor. The only exception is **6 b**, low permeable compound whose transport through membrane seems not to be influenced by P-gp (efflux ratio <2).

## 4. Conclusion

Novel amidino substituted benzimidazole/benzothiazole derivatives **5a**–**5i** and **6a**–**6g** were synthesised and tested for their antiproliferative activity using 2 D and 3 D assays. The compounds were prepared by using conventional synthetic methods. Obtained results revealed that in generaly, the benzothiazole derivatives were more active in comparison to their benzimidazole analogues with the exception of 2-phenyl substituted benzimidazole **6a** which showed enhanced activity, especially for HCC827 cell lines. All active compounds showed strong activity in BrdU assay, in comparison with MTS assay especially for the NCI-H358 cell line.

Additionally, ADME properties of the most active compounds were determined in various *in vitro* assays including solubility, lipophilicity, permeability, metabolic stability and binding to plasma proteins. Five compunds from benzothiazole series (**5c–5e, 5 g, 5 h**) showed moderate to good kinetic solubility, in general good metabolic stability and high binding to plasma proteins. They are also characterised by low permeability and could be classified as P-gp substrates.

Similar ADME properties were obtained for one benzimidazole derivative (**6a**), with the exception of lower lipophilicity and consequently low plasma protein bidning and more pronounced metabolism in liver microsomes. In addition, no significant difference in ADME profile was observed for seven selected non-active compounds.

Tested compouds showed simillar mode of action to the doxorubicin and confirmed obtained low IC50s levels on BrdU assay. Due to promissing antiproliferative effect and lower cell cytotoxicity, described compounds have potential to be part of further developing process for new drugs with antitumor activity and likely with less toxic side effects.
